# A Battle against COVID-19: Vaccine Hesitancy and Awareness with a Comparative Study between Sinopharm and AstraZeneca

**DOI:** 10.3390/vaccines10020292

**Published:** 2022-02-15

**Authors:** Marian S. Boshra, Raghda R. S. Hussein, Marwa Mohsen, Ahmed A. Elberry, Ahmed E. Altyar, Mahmoud Tammam, Rania M. Sarhan

**Affiliations:** 1 Clinical Pharmacy Department, Faculty of Pharmacy, Beni-Suef University, Beni-Suef P.O. Box 62514, Egypt; mariansobhy31@yahoo.com (M.S.B.); raghda.hussien@pharm.bsu.edu.eg (R.R.S.H.); mero_eldawy@yahoo.com (M.M.); 2 Clinical Pharmacy Department, Modern University for Technology and Information, Cairo P.O. Box 12055, Egypt; 3 Clinical Pharmacology Department, Faculty of Medicine, Beni-Suef University, Beni-Suef P.O. Box 62514, Egypt; berry_ahmed@yahoo.com; 4 Pharmacy Practice Department, Pharmacy Program, Batterjee Medical College, P.O. Box 80260, Jeddah 21441, Saudi Arabia; 5 Department of Pharmacy Practice, Faculty of Pharmacy, King Abdulaziz University, P.O. Box 80260, Jeddah 21441, Saudi Arabia; aealtyar@kau.edu.sa; 6 IQVIA, Cairo P.O. Box 10245, Egypt; mahmoud.tammam@hotmail.com

**Keywords:** COVID-19, vaccines, awareness, Sinopharm, AstraZeneca

## Abstract

Background: Awareness about the COVID-19 vaccine’s adverse effects is crucial for gaining public trust. As we still lack proof of vaccines’ safety, this survey aimed to investigate Egyptians’ general awareness of the Sinopharm and AstraZeneca vaccines against COVID-19 and provide considerable evidence on their side effects and complications. Methods: A cross-sectional questionnaire-based study was conducted in Egypt between 20 September and 10 October in 2021, with multiple-choice questions (MCQs) covering all data on vaccine administration confusion, adverse effects or intensity, and complications. Results: Among the 390 participants, 42.3% reported being hesitant before receiving one of the vaccines. About 40.3% of participants were previously infected before getting vaccinated while only 4.6% reported being infected after vaccination. The AstraZeneca vaccine demonstrated higher side effects and symptoms than the Sinopharm vaccine while the Sinopharm vaccine showed a significantly higher rate of COVID-19 infection after vaccination. Conclusions: People with higher educational levels and chronic respiratory diseases represent an excellent model for accepting COVID-19 vaccination. A booster shot is recommended for people vaccinated with the Sinopharm vaccine due to a significantly higher rate of COVID-19 infection after vaccination; however, the Sinopharm vaccine shows a more acceptable safety profile.

## 1. Introduction

On 11 March 2020, the World Health Organization declared the Coronavirus Disease 2019 (COVID-19) pandemic. The global health sectors and economy were challenged by this pandemic [1]. Low- and middle-income countries lacked sufficient medical equipment and essential supplies to cover the increased number of infected people [2].

In the fight against most pandemics, vaccines are the most critical tool. Vaccine hesitancy is a phenomenon that occurs when a considerable number of people worldwide have inquiries about the efficacy, safety, and need for a vaccine [3]. Concerns about vaccines have various dimensions and are influenced by peer pressure [4].

Several factors control the rejection, postponement, or acceptance of vaccination. The decision to manufacture and use a vaccine is dependent on the health institution, political issues, socioeconomics, and culture [5,6]. In early 2020, studies on the intention to get vaccinated against COVID-19 were published and reflected people’s concerns about vaccines’ safety and accessibility [7,8]. The Middle East has been identified as one of the locations with the lowest rates of vaccination adoption worldwide [9].

To develop effective and successful COVID-19 vaccination strategies, it is essential to have a comprehensive understanding of the factors influencing vaccine acceptance and rejection, such as the variability in risk factors and complications that may be associated with various types of vaccines [5,10].

The Sinopharm COVID-19 vaccine, an inactivated coronavirus vaccine developed by Beijing Bio-Institute of Biological Products (BBIBP), is the first Chinese COVID-19 vaccine that the WHO approved for urgent use [11]. The Egyptian Drug Authority (EDA) has approved the Sinopharm Chinese vaccines as the first primary vaccines [12].

Sinopharm has been shown to be safe and well-tolerated in several studies, with 100% of vaccinated patients reporting a robust humoral immune response [13,14]. Furthermore, animal trials on guinea pigs, rats, mice, and rabbits revealed that Sinopharm provided adequate protection against SARS-CoV-2. Nausea, vomiting, fever, dizziness, fatigue, headache, and allergic dermatitis were the most prevalent side effects of the Sinopharm vaccine (which had a 79% efficacy against symptomatic COVID-19 and a 79% efficacy against hospitalization) [15]. Some mild to severe adverse reactions occurred within 28 days of vaccination, with no serious adverse events. Moreover, 2-dose vaccination separated by 1 month produces stronger neutralizing antibody titers than a single 4 or 8 uL dose [16].

The AstraZeneca vaccine, developed by AstraZeneca and COVISHIELD by the Indian Serum Institute (SII), was licensed by WHO for COVID-19. The EUL allows 2 0.5 mL vaccination doses to be administered 4 to 12 weeks apart [17]. Itching, inflammation, blisters, tenderness, discomfort, warmth, and redness at the injection site are all frequent side effects of AstraZeneca, according to the Drugs Controller General of India (DCGI). Aside from arm pain, joint and muscle soreness, general discomfort, lethargy, weariness, chills and fever, headache, and nausea decrease within a few days to a week following immunization [18].

According to the WHO declaration on 19 April 2021, the AstraZeneca vaccine is a successful and safe vaccine with no severe side effects, such as death, hospitalization, or the development of severe diseases [19]. Prophylactic usage of Acetaminophen, according to AstraZeneca Company, can alleviate some symptoms [11].

The present study aimed to analyze the Egyptian population’s awareness of the Sinopharm and AstraZeneca COVID-19 vaccines and to highlight the differences in side effects and consequences.

## 2. Participants and Methods

### 2.1. Participants

The present study assessed the COVID-19 vaccine administration hesitancy, vaccine information, side effects, knowledge, and impact on attitude and expectations. The survey was carried out in Egypt between 20 September and 10 October in 2021 through a Google Form in the form of MCQs, and the data was collected by sharing the survey on social media. The study protocol was approved by the Research and Ethics Committee of Beni-Suef University, Faculty of Pharmacy, Egypt (REC-H-PhBSU-21025) and is in line with the Helsinki declaration. A total of 390 participants were included. All participants were above 18 years old.

### 2.2. Outcomes

All participants’ demographic information was gathered. Participants were asked if they had any chronic illnesses. Hesitation before receiving the vaccine was determined among participants. Participants were asked about the type of received vaccine, either Sinopharm or AstraZeneca. Side effects of both vaccines and their severity were assessed, and if any symptoms appeared after vaccination. Moreover, the medications used to manage post-vaccination symptoms were investigated. If participants developed symptoms, they were asked about the time of its appearance. Furthermore, they were asked about having a D dimer test after receiving the vaccine and if they were infected with COVID-19 after vaccination. Participants were asked about taking anticoagulant medications before vaccination as a precautionary measure. They were asked if vaccination affected the precautionary measures for COVID-19, such as wearing masks and avoiding crowded areas. Knowledge of participants about people who should be prevented from receiving the COVID-19 vaccine and its safety for pregnant and lactating women was determined. Participants were asked about the number of infections before vaccination and the requirement of hospitalization, and post-infection after vaccination. Moreover, they were asked whether the vaccine could help people return to everyday life as before. Participants’ acceptance of giving a vaccine to their children, their history of respiratory disease, and knowledge about preventing certain groups of people from receiving any COVID-19 vaccine were investigated.

### 2.3. Statistical Analysis

#### 2.3.1. Sample Size Calculation

The sample size was calculated using the following formula [20]: $n=Z^{2}\frac{P\left(1-P\right)}{d^{2}}$ assuming an expected outcome (AstraZeneca and Sinopharm Vaccines general knowledge and awareness) of 50% with a margin of error ±0.5% and a confidence level of 95%. The estimated sample size is 384 participants.

#### 2.3.2. Descriptive and Inferential Statistics

The nominal variables in the survey were described using numbers and frequencies. Univariable analysis and associations between key participant characteristics and outcomes were investigated using chi-square or Fisher exact tests, where *p*-values less than 0.05 were recognized as significant

The predictors of COVID-19 infection after vaccination were studied using multinomial logistic regression modeling. Dependent outcomes in the model included if there was a confirmed post-vaccination infection, if the respondent was hesitant about getting infected post vaccination, or if the respondent could affirm the absence of post vaccination infection, which was selected as the referent outcome in the regression model. Patients’ age and gender demographic data, presence of comorbidities, and type of administered vaccine were incorporated as the main predictors in the multinomial logistic regression model. Results are expressed in terms of odds ratio and a 95% confidence interval. IBM SPSS v21 was used for statistical analysis.

## 3. Results

In total, 390 participants were included in the study; the respondents’ demographics are summarized in numbers and percentages in Table 1. In total, 64.9% of the participants were female, 47.7% of the participants were aged between 26 and 35 years, 63.4% were living in urban communities, about 98.5% had pursued a high university educational degree or postgraduate studies, and about 74.4% of the respondents were healthcare workers.

### 3.1. Vaccine Administration Hesitancy

Among the respondents, 42.3% of the respondents reported being hesitant before receiving the vaccine. The determinants of vaccination hesitation are summarized in Table 2. Educational level and presence of chronic respiratory illness were the main determinants of hesitancy, where respondents with high-level education had the lowest hesitancy rate compared to respondents with mid-level education and postgraduate studies, and respondents with a chronic respiratory illness were less hesitant to get vaccinated.

### 3.2. Vaccination Information and Side Effects

About 40.3% of the respondents were previously infected with COVID-19 before getting vaccinated while only 4.6% reported being infected after vaccination. The AstraZeneca vaccination had significantly higher side effects, symptoms, and severity than the Sinopharm vaccine. On the other hand, the Sinopharm vaccine had a significantly higher rate of COVID-19 infections after vaccination.

The multi-nominal regression model including age, gender, presence of chronic illness, and type of administered vaccine was constructed to determine the factors associated with post-vaccination infection, showing a significantly increased risk (OR = 8.39, CI = 1.66–42.23) in patients vaccinated with the Sinopharm compared to the AstraZeneca vaccines.

Common side effects and symptoms experienced from both the Sinopharm and AstraZeneca vaccines are summarized in Table 3. Table 4 shows the perceptions and impact of vaccination on the administration of anti-platelets or anti-coagulants before and after vaccination. None of the respondents were hospitalized when infected prior to vaccine administration.

### 3.3. Vaccination Knowledge and Impact on Attitude and Expectations

Respondents denying or not being sure whether vaccination would decrease infection severity was higher than those in favor of vaccination but with no statistical significance. Most of the respondents showed uncertainty regarding vaccination during pregnancy or lactation. 

Several respondents with higher educational degrees agreed that vaccination would help life to return as regular as before COVID-19, which was statistically significant compared to respondents with mid-level or postgraduate education. Vaccination knowledge, expectations, and attitude impact are summarized in Table 5.

## 4. Discussion

Rejection, hesitancy, rumors, and suspicions have all influenced COVID-19 immunization efforts up to this point. Vaccination apprehension may be influenced by ideas and attitudes regarding COVID-19, such as the impact on one’s life, the virus severity, immunity, and beliefs and attitudes about the vaccine itself, such as the novelty, efficiency, and adverse effects [21].

The current study looked at COVID-19 vaccination intentions in a broad Egyptian population and found that anti-COVID-19 vaccination behavior was highly linked to a lower educational level and the absence of a chronic respiratory condition. These results corroborate other surveys’ findings regarding the impact of educational level on COVID-19 vaccine hesitancy [22,23,24]. An essential finding of this survey was that having certain chronic respiratory illnesses, such as asthma, COPD, or cystic fibrosis, enhances getting vaccinated. Seriously, it was confirmed that patients with chronic respiratory illness were at a high risk of severe forms of COVID-19 and a higher mortality rate compared to patients without a respiratory illness or patients with influenza [25]. In addition, the female gender represented about 60% of the respondents who showed hesitancy towards vaccination, agreeing with previous studies [26,27].

We investigated the safety and effectiveness of the two vaccines independently to identify any differences in reactions or protection against COVID-19. The collection of evidence-based data on local and systemic adverse effects, especially if they are transient or brief, may alleviate worries and encourage completion of the two-dose vaccination series and booster doses in the future [22]. It was found that AstraZeneca (AZ) vaccination had a significantly higher rate of almost all side effects after the first dose than the Sinopharm (SP) vaccine, consistent with the survey conducted by Jordanian healthcare [28]. The majority of side effects recorded after the second dosage of both vaccines were much lower than those reported after the first dose, which was also described in two studies [28,29]. However, after finishing the two-dose vaccination protocol, the Sinopharm vaccine demonstrates a significantly greater rate of COVID-19 infection, showing the vaccine’s low immunogenic potential. Inactivated vaccines, such as Sinopharm, have a high safety profile, but they require a booster shot to establish immunological memory [30]. Meanwhile, the AstraZeneca vaccine was developed through a recombinant vector technique that stimulates the human body to produce higher protection antibodies [31].

There are various adverse reactions following vaccination involving local, such as post-injection pain and numbness and other systemic reactions. Among the systemic side effects, fever represents the most common symptom associated with the AstraZeneca vaccine compared with the SP vaccine, followed by headache and fatigue. Around 7% of respondents who received the AZ vaccine reported the appearance of blue or purple spots on the lower and upper limbs as a symptom of thrombocytopenia compared to 1% of those who received the SP vaccine. In contrast, the analyzed data of the questionnaire administered in Jordon to healthcare workers showed that no severe SE was reported post-AZ vaccination. That may be due to the confounding risk factors that heavily exist in the Egyptian populations. Confounders included stress, binge smoking, presence of comorbidities, anger, fear of getting infected, and exaggerating the severity of vaccine side effects [32]. However, a safety update released by the EMA on 16 April 2021 specified thrombocytopenia as a new common side effect (1 out of 10 individuals) and thrombosis in combination with thrombocytopenia as a unique infrequent side effect (1 out of 10,000 individuals) [33]. While reporting respondents’ answers about performing the d-dimer test after vaccination, they, unfortunately, negated the D-dimer follow-up evaluation (94% of the total respondents). This must be placed under the spotlight to encourage people through vaccine campaigns to adhere more to tests for thrombotic events.

The least common SEs linked with either vaccine were palpitations and shortness of breath, with no significant difference. Gastrointestinal SE was substantially higher in AZ vaccination recipients than in SP vaccine recipients. It could be explained by the vaccine’s nature (S glycoprotein) and its impact on the gastrointestinal tract, previously documented for SARS-CoV-2 [16]. All of these effects were reported to have happened within the first two days of vaccination. Paracetamol was advised as a preventive treatment to decrease the local and systemic effects following vaccination [34].

The level of COVID-19 vaccination acceptance in pregnant women and parents of children under 18 years is an essential subject to be investigated. Estimates of global vaccination acceptance among pregnant women and mothers of small children are unknown as far as we know. Understanding the barriers to vaccination acceptability and the factors that influence vaccine acceptance will help to speed up vaccine administration in these populations [35]. In our poll, most participants (71%) stated that they did not know whether the vaccine was safe for pregnant women while others (16%) said they would not give it to them. A sub-group study of data about whether parents agree that their children should be vaccinated revealed significantly greater acceptance rates among the higher education group (42.3%) than the mid-level education group (0%). It implies that Egyptians are reasonably aware of the importance of childhood vaccination to avoid infection [36,37,38,39,40].

When determining whether vaccination would allow a return to everyday life, it was discovered that respondents with a higher education level agreed with statistical significance that vaccination would assist in returning to everyday life, as opposed to respondents with a mid-level or postgraduate education. Respondents who disputed or expressed doubt that vaccination would reduce infection intensity were higher than those favoring vaccination, but the difference was not statistically significant. As a result, past polls’ findings suggested that vaccination programs and communications should incorporate theoretical frameworks. Contextual factors, such as media coverage of COVID-19 immunization, are likely to encourage vaccination. In addition, the media should clarify and emphasize how immunization might help to prevent the spread of COVID-19 and promote a return to normalcy [21].

## 5. Conclusions

Educational level plays a crucial role in a population’s awareness and acceptability of the COVID-19 vaccination. In addition, patients with chronic respiratory diseases have high acceptability regarding vaccination. The Sinopharm vaccine has weak immunologic activity due to a significantly higher rate of COVID-19 infection after taking the two doses than the AstraZeneca vaccine, so a booster dose is required to activate immunologic memory in people who receive the Sinopharm vaccine. On the other hand, the AstraZeneca vaccine has a significantly higher rate of almost all side effects than the Sinopharm vaccine.

## 6. Limitation of the Study

The number of participants from rural communities was significantly low compared to urban ones. Moreover, the number of participants with mid-level education was significantly low when compared to participants with high-level or postgraduate education. This may be related to the network method of questionnaires, which may be deficient regarding rural area or mid-level education participants. Only 1.8% of the participants were students. This can be attributed to the survey only including participants older than 18 years.

## Figures and Tables

**Table 1:** Participants’ demographics.

Characteristics	No. (%)
Age Group	
18–25 years	76 (19.5%)
26–35 years	186 (47.7%)
36–45 years	86 (22.1%)
46–55 years	16 (4.1%)
more than 55 years	26 (6.7%)
Gender	
Male	137 (35.1%)
Female	253 (64.9%)
Residence Location	
Urban	343 (87.9%)
Rural	47 (12.1%)
Education Level	
Mid-level Education	6 (1.5%)
High-level Education	221 (56.7%)
Postgraduate Education	163 (41.8%)
Profession	
Unemployed	55 (14.1%)
Healthcare Worker	290 (74.4%)
Student	7 (1.8%)
Other Professions	38 (9.7%)
Chronic Illness	
No	335 (85.9%)
Yes	55 (14.1%)

**Table 2:** Vaccination hesitation determinants.

	Non-Hesitant No. = 225 (57.7%)	Hesitant No. = 165 (42.3%)	*p*-Value
Age Group			0.204
18–25 years	48 (63.2%)	28 (36.8%)	
26–35 years	96 (51.6%)	90 (48.4%)	
36–45 years	55 (63.9%)	31 (36.1%)	
46–55 years	11 (68.8%)	5 (31.2%)	
more than 55 years	15 (57.7%)	11 (42.3%)	
Gender			0.662
Male	77 (34.2%)	60 (36.4%)	
Female	148 (65.8%)	105 (63.6%)	
Residence			0.781
Urban	197 (87.6%)	146 (88.5%)	
Rural	28 (12.4%)	19 (11.5%)	
Profession			0.768
Health care worker	170 (75.6%)	120 (72.7%)	
Student	3 (1.3%)	4 (2.4%)	
Other Profession	20 (8.9%)	18 (10.9%)	
Unemployed	32 (14.2%)	23 (13.9%)	
Educational Level			<0.005
Mid-level	1 (0.4%)	5 (3%)	
High-level	146 (64.9%)	75 (45.5%)	
Postgraduate	78 (34.7%)	85 (51.5%)	
Suffer from Chronic Respiratory illness			0.009
No	210 (93.3%)	163 (98.8%)	
Yes	15 (6.7%)	2 (1.2%)	
Suffer from any Chronic illness in General			0.610
Yes	196 (86.7%)	140 (84.8%)	
No	30 (13.3%)	25 (15.2%)	

**Table 3:** Comparison between Sinopharm and Astrazeneca vaccination information, side effects presence, severity, and COVID-19 infection after vaccination.

	Sinopharm No = 197 (50.5%)	Astrazeneca No = 193 (49.5%)	Total No = 390 (100%)	*p*-Value
Have you ever had COVID Infection before receiving the vaccine?				*p* = 0.256
No	86 (43.7%)	99 (51.3%)	185 (47.4%)	
Yes	83 (42.1%)	74 (38.3%)	157 (40.3%)	
Not Sure	28 (14.2%)	20 (10.4%)	48 (12.4%)	
Number of administered doses				*p* < 0.005
Administered 1 dose only	25 (12.7%)	107 (55.4%)	132 (33.8%)	
Administered 2 doses	172 (87.3%)	86 (44.6%)	258 (66.2%)	
Side effects of vaccination				*p* < 0.005
Never had symptoms	78 (39.6%)	15 (7.8%)	93 (23.8%)	
Experienced side effects after the first dose only	63 (32%)	154 (79.8%)	217 (55.6%)	
Experienced side effects after the second dose only	14 (7.1%)	1 (0.5%)	15 (3.8%)	
Experienced side effects after the two doses	42 (21.3%)	23 (11.9%)	65 (16.7%)	
Severity of symptoms				*p* < 0.005
No symptoms	78 (39.6%)	15 (7.8%)	93 (23.8%)	
Mild	88 (33.7%)	41 (21.2%)	129 (33.1%)	
Moderate	27 (13.7%)	81 (42%)	108 (27.7%)	
Severe	4 (2%)	56 (29%)	60 (15.4%)	
Duration till having symptoms				*p* < 0.005
No symptoms	78 (39.6%)	15 (7.8%)	93 (23.8%)	
On the first day after taking the vaccine	65 (33%)	108 (56%)	173 (44.4%)	
On the second day after taking the vaccine	33 (16.8%)	65 (33.7%)	98 (25.1%)	
Several days after receiving the vaccine	21 (10.7%)	5 (2.6%)	26 (6.7%)	
Used medications to manage post-vaccination symptoms				*p* < 0.005
No	124 (62.9%)	44 (22.8%)	168 (43.1%)	
Yes, paracetamol	55 (27.9%)	131 (67.9%)	186 (47.7%)	
Yes, an anti-inflammatory drug	18 (9.1%)	18 (9.3%)	36 (9.2%)	
Performing D-dimer test after Vaccination				*p* = 0.332
No	186 (94.4%)	182 (94.3%)	368 (94.4%)	
Yes, and it was elevated	2 (1%)	0 (0%)	2 (0.5%)	
Yes, and it was normal	9 (4.6%)	11 (5.7%)	20 (5.1%)	
Got COVID-19 Infected after Vaccination				*p* < 0.005
No	145 (73.6%)	177 (91.7%)	322 (82.6%)	
Yes	16 (8.1%)	2 (1%)	18 (4.6%)	
Not Sure	36 (18.3%)	14 (7.3%)	50 (12.8%)	
Was you infected with COVID-19 after receiving the first dose of the vaccine?				*p* = 0.332
No	185 (93.9%)	180 (93.3%)	365 (93.6%)	
Yes	5 (2.5%)	2 (1%)	7 (1.8%)	
Not sure	7 (3.6%)	11 (5.7%)	18 (4.6%)	
Was you infected with COVID-19 after receiving the second dose of the vaccine?				*p* < 0.005
No	156 (79.2%)	191 (99%)	347 (89%)	
Yes	11 (5.6%)	0 (0%)	11 (2.8%)	
Not Sure	30 (15.2%)	2 (5.2%)	32 (8.2%)	
Have you confirmed that you or any of those around you have Pulmonary Embolism?				*p* = 0.007
No	197 (100%)	186 (96.4%)	383 (98.2%)	
Yes	0 (0%)	7 (3.6%)	7 (1.8%)	

**Table 4:** Comparison between the Sinopharm and Astrazeneca vaccines’ common side effects.

	Sinopharm No = 197 (50.5%)	Astrazeneca No = 193 (49.5%)	Total No = 390 (100%)	*p*-Value
Administration of blood thinning medication before vaccination as a precautionary measure				*p* = 0.005
No	188 (95.4%)	169 (87.6%)	357 (91.5%)	
Yes	9 (4.6%)	24 (12.4%)	33 (8.5%)	
Did taking the vaccine affect your regularity in taking blood thinners?				*p* = 0.008
No	195 (99%)	180 (93.3%)	375 (96.2%)	
Yes	2 (1%)	6 (3.1%)	8 (2.1%)	
Not Sure	0 (0%)	7 (3.6%)	7 (1.8%)	

**Table 5:** Vaccination knowledge, expectations, and attitude impact.

	Mid-Level Education	High-Level Education	Postgraduate Education	Total
Does the vaccine affect the severity of the disease?				*p* = 0.196
No	3 (50%)	148 (67%)	92 (56.4%)	243 (62.3%)
Yes	0 (0%)	17 (7.7%)	17 (10.4%)	34 (8.7%)
Don’t Know	3 (50%)	56 (25.3%)	54 (33.1%)	113 (29%)
Is the vaccine safe for pregnant and lactating women?				*p* = 0.342
No	0 (0%)	37 (16.7%)	26 (16%)	63 (16.2%)
Yes	0 (0%)	32 (14.5%)	16 (9.8%)	48 (12.3%)
Don’t Know	6 (100%)	152 (68.8%)	121 (74.2%)	279 (71.5%)
Would you agree to your children below 18 taking the vaccine?				*p* = 0.028
No	2 (33.3%)	79 (35.7%)	45 (27.6%)	126 (32.3%)
Yes	0 (0%)	94 (42.5%)	69 (42.3%)	163 (41.8%)
Don’t Know	4 (66.7%)	48 (21.7%)	49 (30.1%)	101 (25.9%)
Did the vaccine reduce your precautionary COVID measures (wearing masks & Avoidance of crowded places)?				*p* = 0.009
No	2 (33.3%)	138 (62.4%)	95 (58.3%)	235 (60.3%)
Yes	0 (0%)	54 (24.4%)	40 (24.5%)	94 (24.1%)
Don’t Know	4 (66.7%)	29 (13.1%)	28 (17.2%)	61 (15.6%)
Would you agree to your children below 18 taking the vaccine?				*p* = 0.028
No	2 (33.3%)	79 (35.7%)	45 (27.6%)	126 (32.3%)
Yes	0 (0%)	94 (42.5%)	69 (42.3%)	163 (41.8%)
Don’t Know	4 (66.7%)	48 (21.7%)	49 (30.1%)	101 (25.9%)
